# Comprehensive Analysis of a tRNA-Derived Small RNA in Colorectal Cancer

**DOI:** 10.3389/fonc.2021.701440

**Published:** 2021-08-04

**Authors:** Yong Zhu, Shaoqiu Chen, Zhougui Ling, Andrew Winnicki, Lilly Xu, Shaun Xu, Ting Gong, Bin Jiang, Gang Huang, Youping Deng

**Affiliations:** ^1^National Medical Centre of Colorectal Disease, The Third Affiliated Hospital of Nanjing University of Chinese Medicine, Nanjing, China; ^2^Department of Cell & Molecular Medicine, Rush University Medical Center, Chicago, IL, United States; ^3^Department of Quantitative Health Sciences, John A. Burns School of Medicine, University of Hawaii at Manoa, Honolulu, HI, United States; ^4^Molecular Biosciences and Bioengineering Program, College of Tropical Agriculture and Human Resources, University of Hawaii at Manoa, Honolulu, HI, United States; ^5^Department of Pulmonary and Critical Care Medicine, The Fourth Affiliated Hospital of Guangxi Medical University, Liuzhou, China; ^6^Shanghai Key Laboratory for Molecular Imaging, Shanghai University of Medicine and Health Sciences, Shanghai, China

**Keywords:** colorectal cancer, tRNA-derived small RNAs, diagnosis, prognosis, random forest

## Abstract

Colorectal cancer often presents as a highly variable disease with myriad forms that are at times difficult to detect in early screenings with sufficient accuracy, for which novel diagnostic methods are an attractive and valuable area of improvement. To improve colorectal cancer diagnosis and prognosis, new biomarkers that can be assembled into a diagnostic panel must be identified, and tRNA-derived small RNAs (tsRNAs) are a particularly interesting and increasingly visible new class of molecules to examine. In this study, small RNA-seq data were profiled for the expression of 104 human tsRNAs in tumor tissue and adjacent normal tissue samples, and a diagnostic model was built based on four differentially expressed tsRNAs: tRF-22-WB86Q3P92, tRF-22-WE8SPOX52, tRF-22-WE8S68L52, tRF-18-8R1546D2. Furthermore, the diagnostic model was validated by two independent validation datasets (AUC was 0.97 and 0.99), and a LASSO model was applied to develop a seven-tsRNA-based risk score model for colorectal cancer prognosis. Finally, a tsRNA-mRNA interaction network was established according to potential mRNA targets predicted by bioinformatic methods. In conclusion, the results suggest that abnormal expression of tsRNA in colorectal cancer may have a functional effect on tumor action and moreover, that some of the tsRNAs identified in this study with diagnostic and prognostic potential could be of clinical significance.

## Introduction

Colorectal cancer (CRC) is currently one of the most common types of cancers in the world, with incidence and mortality rates increasing over the last 25 years in adults under the age of 50 (1–3). In both sexes combined, CRC accounts for the third highest rate of cancer incidence at 10.2% cases and the second highest mortality rate at 9.2% (3–5). According to the WHO, in 2018, more than 1.8 million new cases of CRC were diagnosed worldwide, with 862,000 deaths, and the global burden is expected to balloon to 2.2 million new cases and 1.1 million deaths by 2030 (4). Moreover, given the growth and aging of the population, the global burden of CRC can only be expected to increase further, including in developing countries where the increasing rate of incidence is aggravated largely by late presentation and inaccessibility to early screening. CRC is not isolated to developing countries, but on the contrary is an issue much more pronounced in high HDI countries: for example, according to the CDC, in the United States alone, 141,270 new CRC cases emerged in 2016 with 52,286 deaths (5). However, despite these harrowing statistics, colorectal cancer is also one of the most preventable types of cancer with a high survival rate after early detection. The efficacy of early detection and prompt treatment of the disease is demonstrated markedly in developed countries, where early screening rates have escalated over the last decade and mortality has been on a largely downward trend overall (6–8). For example, patients who participated in flexible sigmoidoscopy for screening of CRC have shown significantly reduced incidence and mortality rates that were sustained over the long term, but the US Preventative Services Task Force still reports a lack of definitive evidence for a single-best screening approach, and current methods for early detection such as the endoscopy are typically fairly invasive (9, 10). The development of noninvasive, accurate, and accessible data-driven methods might contribute significantly to the establishment of socially normalized, regular CRC screenings for millions of adults around the world. Furthermore, significantly reducing barriers to access early screening methods would go a long way to preventing developing countries from inheriting the issues that accompany shifting diets and lifestyles, as well as perhaps even attenuate the asymmetric development of CRC along socioeconomic lines (1–3). This issue is, therefore, of great urgency, and the confluence of problems that CRC presents our society is highly multidimensional. Indeed, in recent years, the CDC has engaged in enormous movements to encourage increased CRC screening rates to at least 80% in every community by reducing screening barriers (5). Thus, the need for new methods of detecting CRC early presents itself both as a burgeoning need in developed countries as well as a new area of demand in developing countries with the potential for tremendous positive impact.

This study explores the potential of tRNA-derived small RNA (tsRNA) to address this immense need. In recent years, the study of small non-coding RNAs (ncRNAs) has expanded rapidly to become one of the hottest areas of research in the field with the revelation of their biological and even clinical significance. Many ncRNAs have been shown to play key roles in regulating cancer and are associated with a variety of biological processes, and moreover some are stable in the bloodstream where they could be used as the basis for a cancer screening with only a few drops of blood (11–14). Thus, ncRNAs are an alluring target for active translation into the clinic. In particular, tsRNAs are a novel type of regulatory small non-coding RNA that has attracted the attention across multiple subfields of biology both as an area that appears fertile for development of new early detection methods, and also for the allusion in recent studies to its participation in a variety of biological processes under diverse pathological and physiological conditions (15–18). Today, different forms of tsRNA are known to emerge from cleavage of tRNA or pre-tRNA at well-defined sites, and that they are not simply a degradation product but rather one that is precisely regulated (11). Moreover, recent research has shown that tsRNA disorders are associated with a number of different cancers as either oncogenic factors or tumor suppressors, and that their expression is related to cancer development and staging (19). In a recent study, tsRNAs were found to be upregulated in liver tumors, and even more importantly, the targeting of these tsRNAs with an oligonucleotide resulted in cell death both *in vitro* and *in vivo* (20). Additionally, in liver cancer, regulation of tsRNA expression was found to be associated with overall survival of the disease (21). Taken together, these important results indicate the potential clinical and biological importance of tsRNA, yet they also highlight the vast space for future research to improve our understanding of these ncRNAs. There are many questions about the biogenesis of tsRNA and their precise roles and mechanisms within the body that remain to be illuminated, and whether the expression profile of tsRNAs could present a stable bloodborne signal representative of the internal tumor state for use in a clinical setting. Research in tsRNAs promises exciting new applications and discoveries, and efforts to elucidate their poorly understood background and clinical significance represent an important and emerging facet of cancer research at large. tsRNAs are a prime candidate for further examination towards achieving the goal of addressing the immense needs for easily accessible, non-invasive, accurate early CRC screening methods described, and even as a potential therapeutic target.

## Methods

### Dataset and Small RNA Sequence Processing

Discovery of tsRNAs and the independent validation set 1 were obtained from the Sequence Read Archives (SRA) public repository (SRP107326, SRP183064). The independent validation set 2 and prognostic dataset were downloaded from the TCGA data portal (https://tcga-data.nci.nih.gov/tcga). All small RNA-seq were aligned using sRNAtools software to obtain tsRNA expression levels. The full details of this method have been published previously (22). Briefly, all miRNA-seq FASTQ files were con-verted to collapsed FASTA format after removal of adapters. The TCGA miRNA-seq BAM files are files that have removed adapters. Unprocessed FASTQ format reads had adapters trimmed and filtered for 16 nucleotides utilizing Cutadpt 2.1. Missing values was imputed by MetImp 1.2 (23).

### Statistical Analysis

Adjacent normal tissue and tumor tissue were analyzed using the paired student t-test in building the diagnostic model, and statistically significant tsRNA sequences were selected. The diagnostic model was constructed using a random forest classifier on these selected tsRNA markers to differentiate colorectal cancer patients from controls. The Kaplan-Meier (KM) test was used for the analysis of the prognostic survival curve and the log-rank test to quantify the survival curve. The Cox regression model was applied in multivariable survival analysis. All analyses were implemented in R software (version 4.0.1). P-values less than 0.05 were statistically significant.

### Target Prediction and GO Analysis

The RNAhybrid (https://bibiserv.cebitec.uni-bielefeld.de/) algorithm was used to predict the potential binding targets of mRNAs indicated by significant prognostic tsRNAs (screening rules: energy < -25 kcal/mol). GO analysis (www.geneontology.org/) was used to identify the predicted target genes, and all involved GO genes were assessed. Fisher’s test was used to calculate the significance level (p-value) of every GO gene, and the outcomes were adjusted using the FDR method in multiple hypothesis testing to classify statistically significant GO genes from differential significant gene enrichment. The network diagram was visualized with Cytoscape software (version 3.5.1).

### Pathway Analysis

The statistically significant tsRNAs’ target gene enrichment analysis was performed by the KEGG pathway (www.kegg.jp/). The hypergeometric distribution of Fisher’s test informed calculation of all pathway weights (p-value) and was adjusted by the FDR. ShinyGo (http://bioinformatics.sdstate.edu/go/) was used to analyze biological process, cellular components, and identify molecular function in conjunction with Pfam and InterPro databases.

## Results

### Altered Expression Profiles of tsRNAs

**Figure 1:**
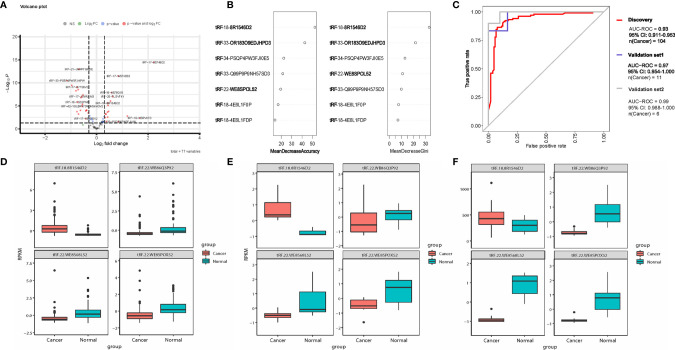
tsRNA diagnostic panel. **(A)** Volcano plot of differentially expressed tsRNAs between normal and cancer. **(B)** Random forest RF mean decrease of accuracy and mean decrease Gini score rank. **(C)** ROC curve of the diagnostic prediction model with tsRNA markers in the discovery data and two independent validation data sets. **(D–F)** Four model selected tsRNA expression in discovery dataset and two independent validation data sets.

**Supplementary Figure 1** displays the data processing workflow. **Table 1** shows the clinicopathological characteristics of the three cohorts that were analyzed through small RNA-seq. In the discovery dataset, 104 paired colorectal cancer samples with adjacent normal tissue samples were analyzed by small RNA-seq and differentially expressed tsRNAs were extracted for both tumor and normal tissue (**Figure 1A**). Significantly expressed tsRNAs were selected (p<0.05, |log2 Fold change| > 1) to build the diagnostic model (**Supplementary Table 1**). The nomenclature contrasts the Mintbase naming method to avoid confusing labels in distinct databases.

**Table 1:** Demographic and clinical characteristic of patients with Colorectal cancer in discovery Cohort set and two independent validation Cohort.

Cohort	SRP107326	TCGA	SRP183064
**Num. of patients**			
Adjacent Normal	104	11	6
Primary Tumor	104	603	6
**Age in years, median (IQR)**	62(55.7-68.2)	67.0(57.0-65.8)	
**Sex**			
Male, count (%)	59(56.7)	315(52.2)	
**Race**			
Asian	104	13	6
White		308	
Black or African American		71	
American Indian or Alaska Native		1	
Not Reported		210	
**Treatment Type**			
Radiation Therapy	Not Reported	307	Not Reported
Pharmaceutical Therapy		296	
**Stage**			
Stage I	18	104	
Stage II	29	180	
Stage III	42	172	
Stage IV	13	89	
Not Reported		24	6
**Primary Diagnosis**			
Adenocarcinoma		501	
other		68	
**Events**			
Death	Not Reported	478	Not Reported
Alive		125	
**Follow Up time (days, IQR)**		638.0(370.5-1091.0)	

### Multi-tsRNAs Diagnostic Model

All 52 tsRNAs with dysregulated expression were selected for features in the random forest (RF) model. According to the RF mean decrease of accuracy and mean decrease in Gini entropy, four significant tsRNAs were picked: tRF-22-WB86Q3P92, tRF-22-WE8SPOX52, tRF-22-WE8S68L52, tRF-18-8R1546D2 (**Figure 1B**). Model performance was evaluated by testing out-of-bag (OOB) samples. We assessed the model performance by receiver operating characteristic curve (ROC curve). The four-tsRNA-based model has AUROC 0.93 (**Figure 1C**) in the discovery dataset. Two independent datasets were then applied to test the classification model. Based on the OOB predicted probabilities (Prob.), a diagnostic RF-score was calculated for the two independent validation datasets. The area under the receiver operating characteristic curve (AUC) was 0.97 and 0.99 (**Figure 1C**). **Figures 1D–F** shows the expression levels of the 4 tsRNAs in the three data sets.

### Multi-tsRNA Based Prognostic Model

**Figure 2:**
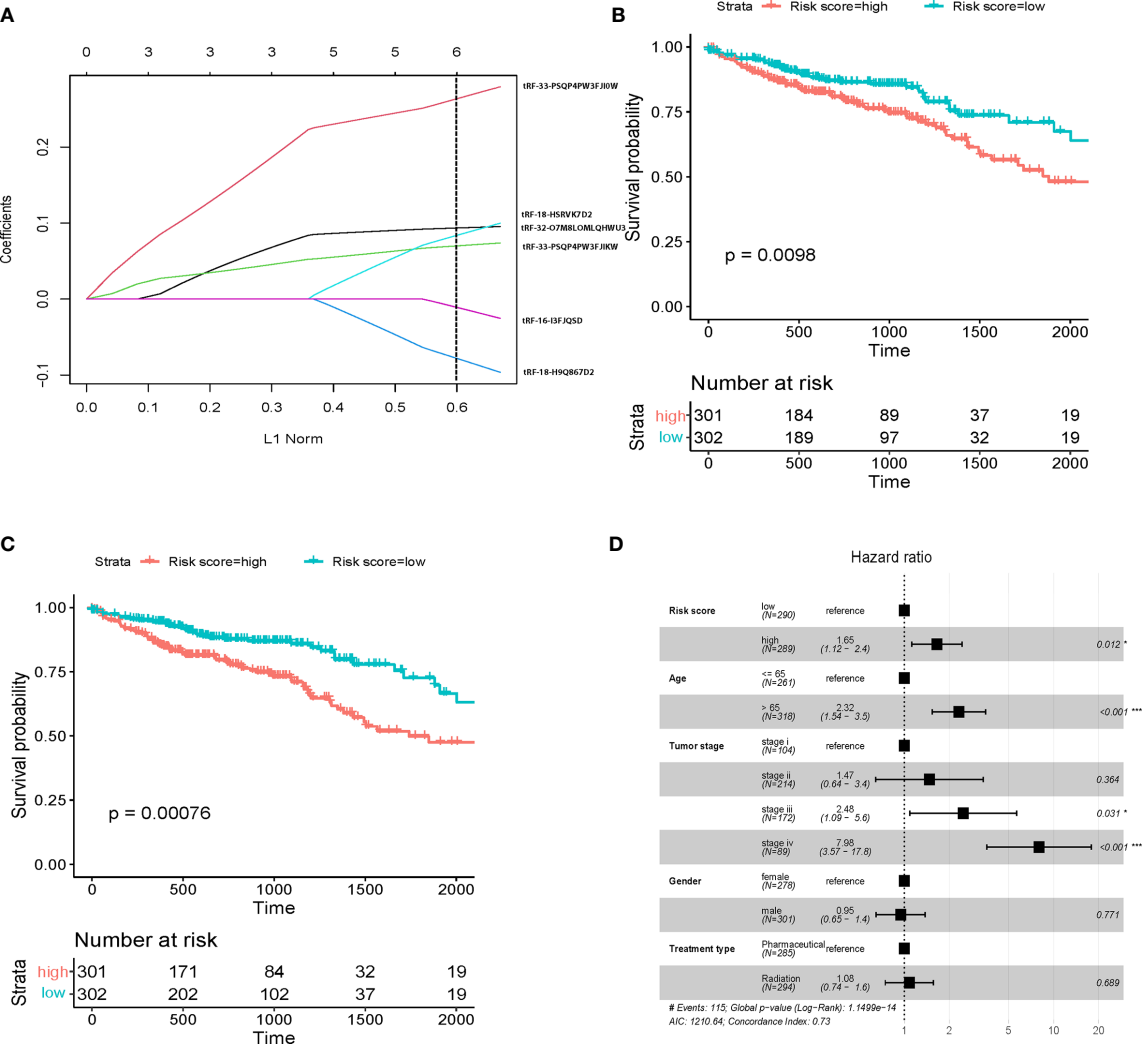
tsRNA prognostic panel. **(A)** prognostic marker selection: Univariant-Cox and LASSO-Cox were applied to a cohort with survival data to identify seven markers’ final determination. L1 norm is LASSO coefficient profiles of the 8 tsRNAs. A vertical line is drawn at the optimal value by 1 s.e. criteria and results in six non-zero coefficients. Five tsRNAs were selected in the LASSO Cox regression model. **(B)** Kaplan-Meier survival curve showing overall survival (OS) in patients without clinical characteristic in high risk and low risk score; **(C)** Kaplan-Meier survival curve showing overall survival (OS) in patients with clinical characteristic in high risk and low risk score; **(D)** Multivariate Cox regression analysis of the tsRNA-based prognostic model with OS. Event; *p < 0.05; ***p < 0.001.

The expression data of 603 colorectal cancer patients informed the prognostic model from the TCGA dataset. A univariate Cox regression analysis was first performed of the overall survival based on each tsRNA. We further utilized a LASSO Cox regression model (**Figure 2A**) to build a prognostic model, including six of the seven significant tsRNAs: tRF-18-HSRVK7D2, tRF-33-PSQP4PW3FJI0W, tRF-33-PSQP4PW3FJIKW, tRF-18-H9Q867D2, tRF-32-O7M8LOMLQHWU3, and tRF-16-I3FJQSD. We estimated the risk score for each patient according to individualized values of six tsRNAs: risk score = (0.094 x tRF-18-HSRVK7D2) + (0.268 x tRF-33-PSQP4PW3FJI0W) + (0.07 x tRF-33-PSQP4PW3FJIKW) -(0.08x tRF-18-H9Q867D2) + (0.08 x tRF-32-O7M8LOMLQHWU3) -(0.01x tRF-16-I3FJQSD). **Figure 2B** shows the disease-free survival curves of high-risk patients and low-risk patients (cutoff=-0.1). Low-risk patients have a better possibility of survival than high-risk patients (p<0.01).

### Multivariable Analysis

**Figure 3:**
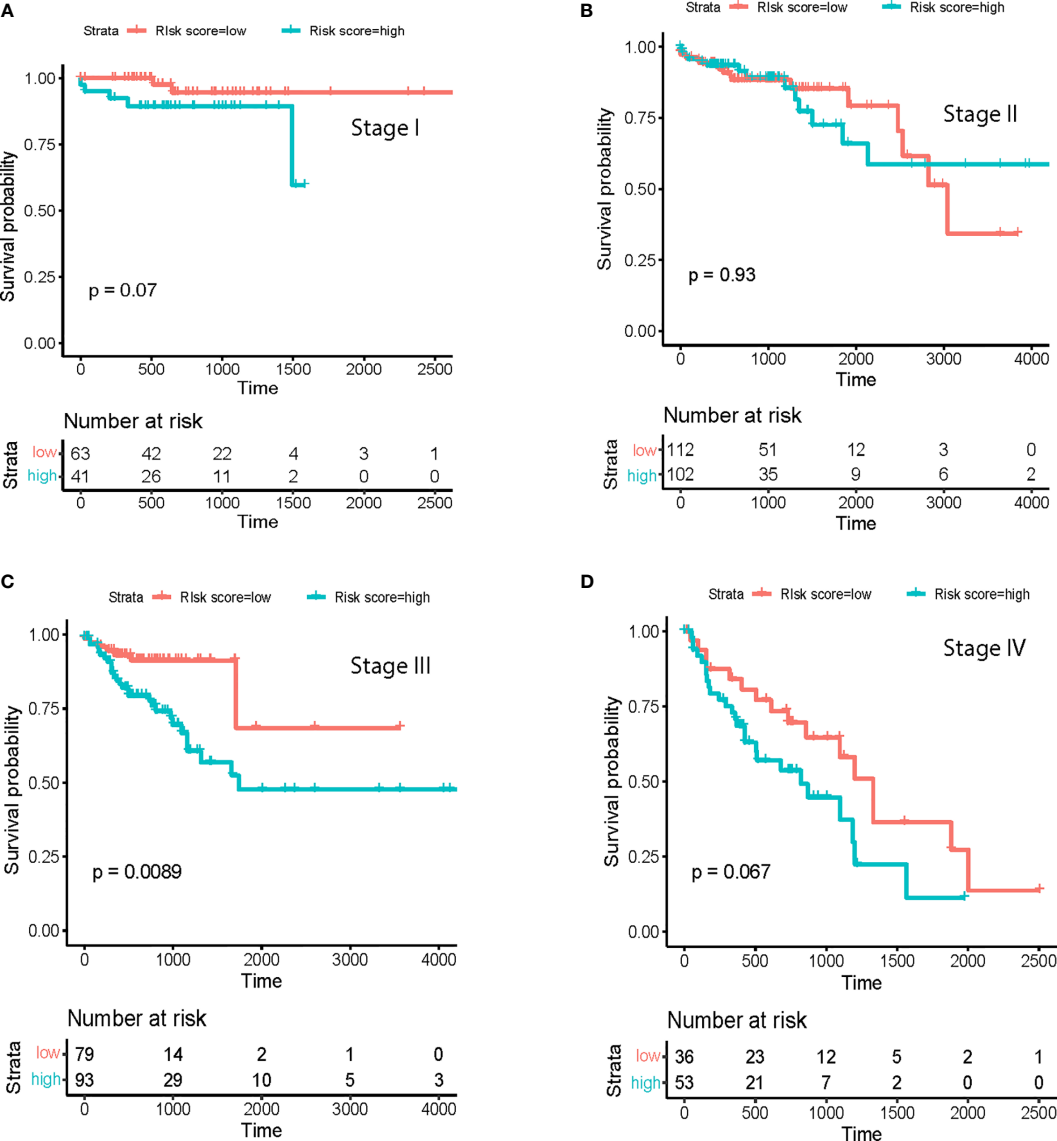
Survival analysis at different cancer stages. Prognostic model in **(A)** Stage I; **(B)**, Stage II; **(C)**, Stage III and **(D)** Stage IV.

To further establish the prognostic significance of tsRNAs in colorectal cancer, we also deployed the prognostic model in combination with other clinicopathological characteristics of colorectal cancer patients from the TCGA database (**Figure 2C**, p<0.001). Importantly, the previously identified association was maintained even after multivariate adjustment for clinicopathological characteristics [p<0.05, HR 1.65(1.12-2.4)] (**Figure 2D**). These outcomes indicated the utility of a combination of our predictive tsRNAs and clinical factors towards improving predictive prognosis accuracy. Moreover, survival curves were drafted according to the Kaplan-Meier method for the patients with risk score in different cancer stages (**Figures 3A–D**) and tumor stages (**Supplementary Figure 2**). However, prediction of the risk score in stage II was poor.

### Bioinformatic Prediction

**Figure 4:**
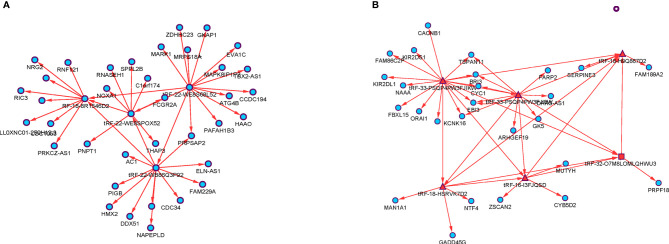
The tsRNA/mRNA network analysis. **(A)** Four diagnostic model selected tsRNAs and their predicted target mRNAs. **(B)** Seven prognostic model selected tsRNAs and their predicted target mRNAs.

The significantly differentially expressed tsRNAs in the diagnostic and prognostic model were investigated using bioinformatic methods. First, the current understanding is that tsRNAs can achieve a miRNA-like form of action, interacting with mRNA targets to regulate gene expression. The interactions between tsRNAs and mRNA transcripts were predicted by the prediction tool RNAhybrid, including the binding sites’ location on the target, the critical region, and free energy of the imperative stability with graphic illustration. As a result, a tsRNA-mRNA interaction network was built using the mRNA associated with each tsRNA, identified according to the prediction score (**Figure 4**).

**Figure 5:**
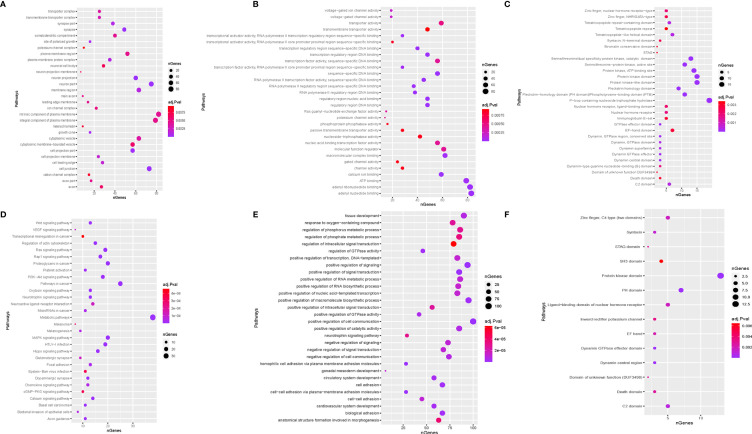
Function enrichment analysis. **(A)** biological process; **(B)** cellular component; **(C)**, molecular function; STRING-db enrichment in **(D)** Pfam; **(E)** KEGG; **(F)** interPro enrichment.

Second, a bioinformatic analysis was performed on all predicted target gene functions using GO analysis, KEGG pathway, and STRING-db enrichment. GO analysis comprises three domains of exploration: biological processes, cellular components, and molecular functions. STRING-db enrichment covers Pfam and interPro enrichment. **Figure 5** shows that the most significant enrichment and the most meaningful terms identified were the intrinsic component of plasma membrane, adenyl nucleotide binding, and positive regulation of cell communication in cellular components, molecular function, and biological processes (**Figure 5**).

Base on the KEGG enrichment analysis and STRING-db enrichment pathways in cancer, protein kinase domain and P-loop containing nucleoside triphosphate hydrolase were significantly identified for each candidate tsRNA (**Figure 5**).

## Discussion

The Human Genome and ENCODE Projects have shown that non-coding RNAs occupy more than 90% of the entire genome (24). Unlike DNA, non-coding RNA is more movable, flexible, and self-folds into a distinct structure that interacts with DNA, RNA, or protein (25, 26). Over the last several years, miRNA, lncRNA, snoRNA, and snRNA have been studied widely (27, 28). However, tsRNA, a new type of ncRNA, has not been studied extensively, especially with respect to cancer occurrence, progression, and metastasis.

This study developed and validated a novel diagnostic and prognostic tool based on tsRNAs to improve colorectal cancer diagnosis and disease recurrence prediction. Our results revealed that the diagnostic model could successfully classify patients into cancer and normal populations. The predictive model could also categorize patients into high-risk and low-risk groups with significant differences in 5-year disease-free survival. Despite the significant performance of tsRNAs in a CRC diagnostic model, the prognosis for patients with CRC at large remains poor. Moreover, the predictive model is inferior in CRC patients with stage II. Approximately one-quarter of CRC patients are diagnosed with stage II. While beneficial for stage III patients, the role of standard treatment is unestablished in stage II disease. At present, it is not possible to precisely differentiate between high-risk and low-risk prognoses for stage II patients (29, 30). Thus, there is an urgent need to explore additional prognostic markers to inform treatment plans for stage II CRC patients.

In functional analysis of the tsRNAs, there is mounting evidence that tRFs have been related to acting as functional regulatory factors in immunity, physiological processes, and cellular metabolism (31, 32). Furthermore, some tsRNAs are dysregulated in various cancers, and that their expression is variable during cancer developing and staging (21, 33, 34). Notably, unusual tsRNAs are identified to bind to mRNA targets like the canonical miRNAs (35, 36). According to such developments, potential target-binding mRNAs for dysregulated tsRNAs were predicted and refined to build tsRNA-mRNA interactions for functional analysis. Predicted genes in STRING-db enrichment, cancer, Protein kinase domain, and P-loop containing nucleoside triphosphate hydrolase were the top signaling pathways affected by the dysregulated tsRNA-mRNA interaction. These enriched pathways may be the targets of the significant tsRNAs, presenting intriguing and potentially valuable avenues for future study.

Although models were established for the diagnosis and prognosis of CRC and the functions of these tsRNAs were predicted, questions that require further investigation still remain. Also, the diagnostic model built by cancer and adjacent normal is not as valuable as the prognostic model. Further prospective, large-scale, multicenter case-control studies are warranted to validate our results and identify the risk factors of CRC. In the predictive model, patients with CRC stage II need to be studied separately, and a new predictive model should be established. Moreover, further *in vitro* experimental verification is required to establish the predicted target mRNAs.

## Data Availability Statement

The original contributions presented in the study are included in the article/**Supplementary Material**. Further inquiries can be directed to the corresponding authors.

## Ethics Statement

The studies involving human participants were reviewed and approved by TCGA. The patients/participants provided their written informed consent to participate in this study.

## Author Contributions

YD conceptualized the idea and launched the study. YZ, ZL, and SC gathered and processed data. YZ, SC, and AW drafted the manuscript. LX, TG and SX was responsible for proofreading. All authors contributed to the article and approved the submitted version.

## Funding

This work was also supported by NIH Grants P30GM114737, P20GM103466, P30CA071789, and U54MD007601.

## Conflict of Interest

The authors declare that the research was conducted in the absence of any commercial or financial relationships that could be construed as a potential conflict of interest.

## Publisher’s Note

All claims expressed in this article are solely those of the authors and do not necessarily represent those of their affiliated organizations, or those of the publisher, the editors and the reviewers. Any product that may be evaluated in this article, or claim that may be made by its manufacturer, is not guaranteed or endorsed by the publisher.
