# Supplementary material for: Comprehensive Analysis of a tRNA-Derived Small RNA in Colorectal Cancer
Source: Front Oncol. 2021 Aug 4;11:701440. doi: 10.3389/fonc.2021.701440 (PMC8371552; doi:10.3389/fonc.2021.701440)
Supplement: Supplementary file 1 [file DataSheet_1.pdf]

Supplementary Appendix

Comprehensive analysis of a tRNA-Derived Small RNA in Colorectal Cancer

Supplementary Figure S1. Workflow of data process. tsRNA expression levels on GEO paired colon cancer and normal were used to identify candidate biomarkers(Left panel). Right panel: diagnostic marker selection, random-forest analyses were used to a discovery data to identify a final determination of four markers. These four markers were applied to two independent validation cohort. Prognostic marker selection, Univariant-Cox and LASSO-Cox were applied to a TCGA cohort with survival data to identify markers' final determination. These markers were used to function prediction tsRNA-miRNA network and pathway analysis.

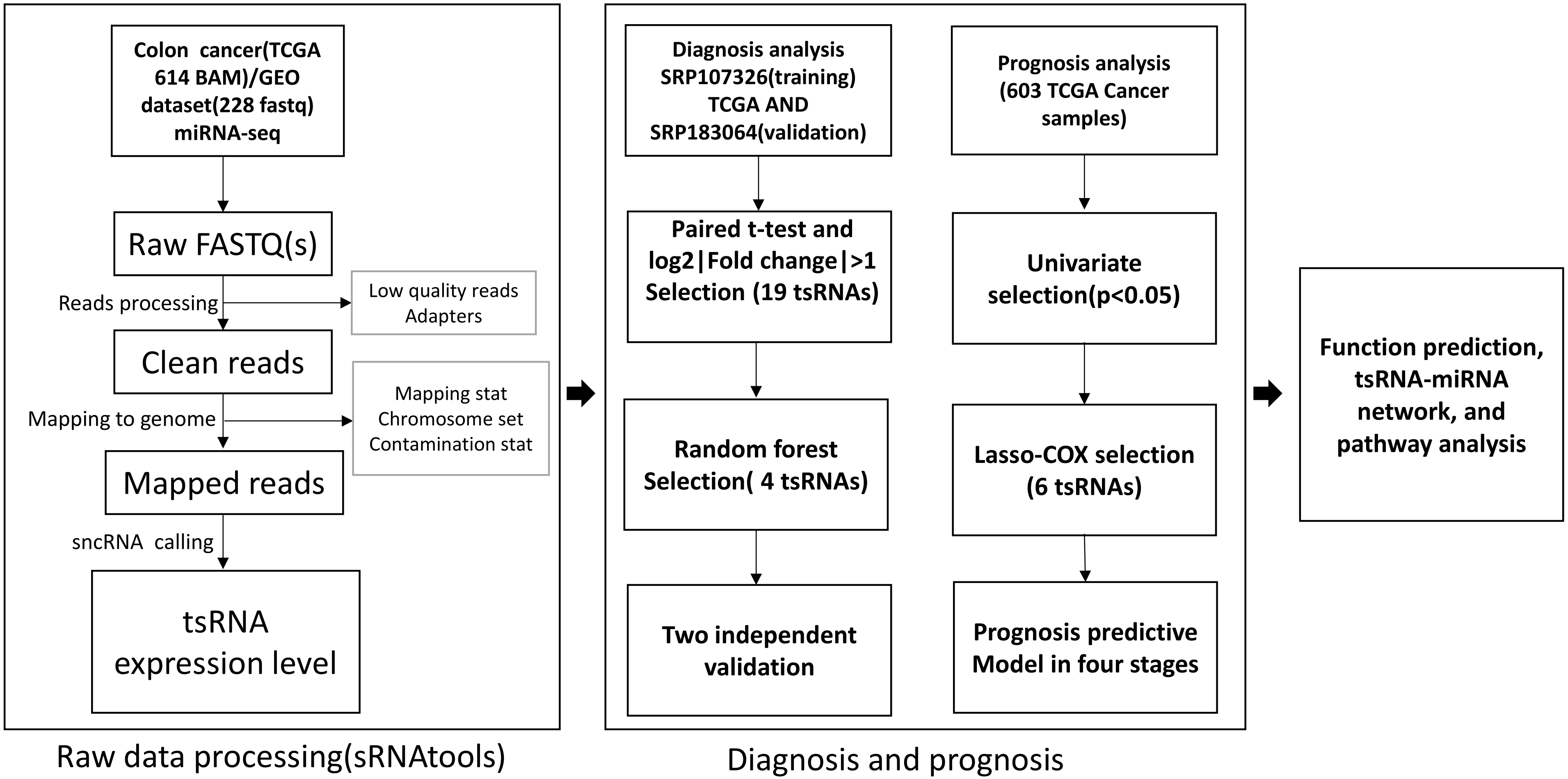

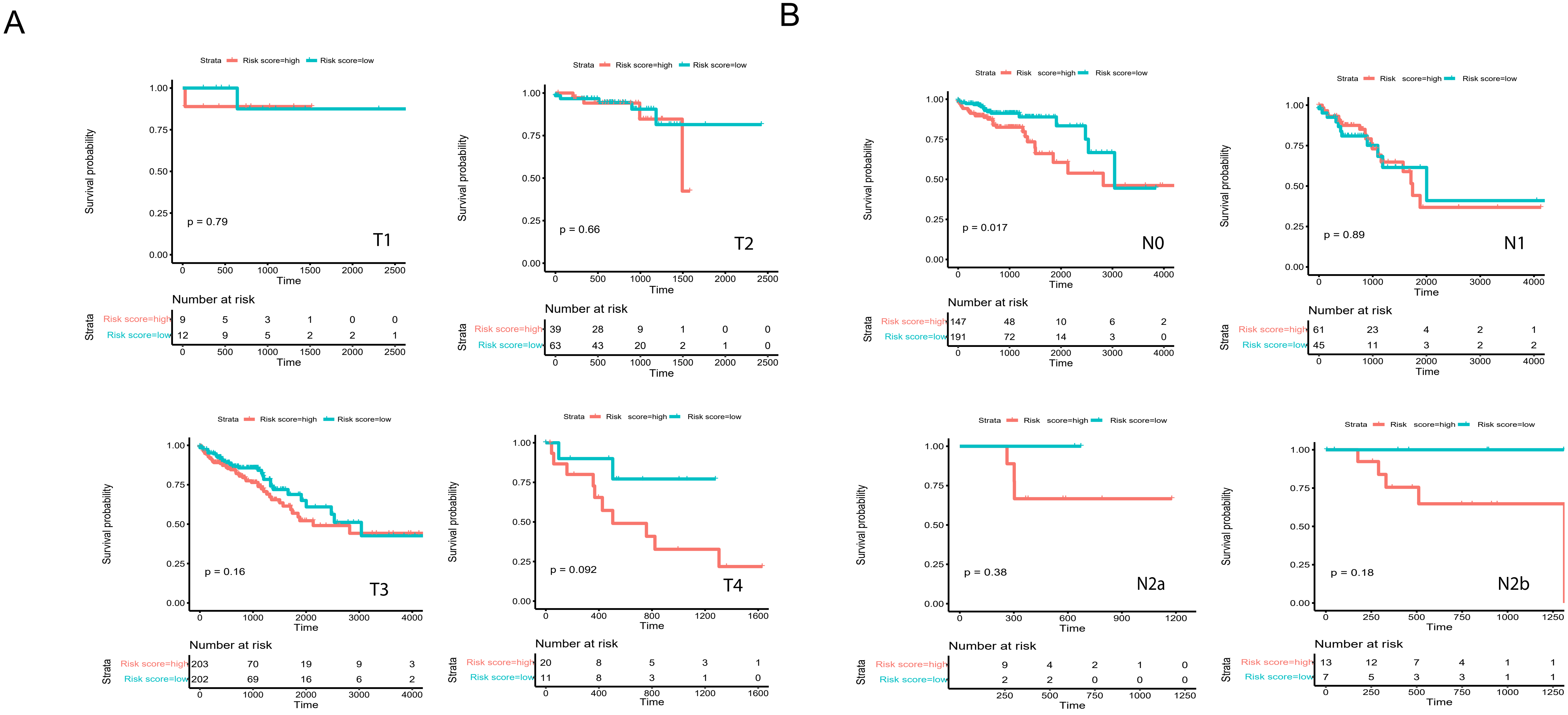

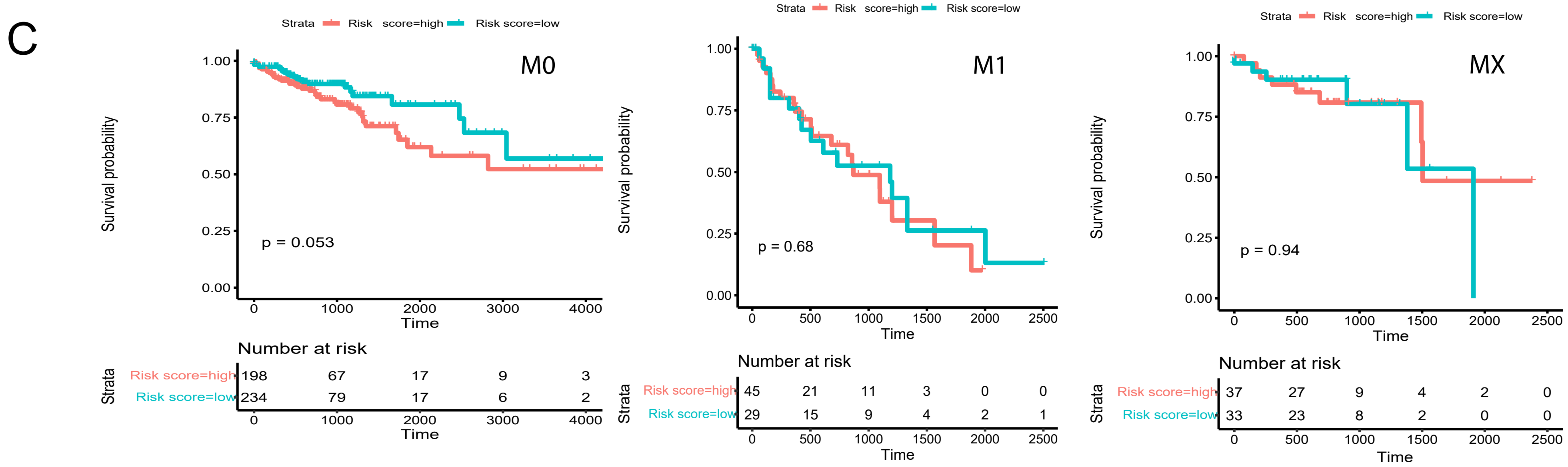

Supplementary Figure S2. Survival analysis at different tumor stages.Prognostic model in A, Primary tumor (T); B, Regional lymph nodes (N); C, Distant metastasis (M).

Supplementary Table 1. A list of tsRNAs that p-value less than 0.05 with tRF sequences when the paired student t-test was evaluated.

ID	Sequence	p Values	Fold Change (C/N)	FDR
var_60	TCCCGGTGTGGGAACCA	3.67E-15	4.663809097	2.86E-13
var_47	CAGTCGGTAGAGCATGGGACT	1.09E-13	0.621708431	4.27E-12
var_37	TCTCGGTGGGACCTCCA	3.28E-12	1.79950102	8.54E-11
var_109	GCCCGGATAGCTCAGTCGGTAGAGCATCAGACT	3.48E-11	0.45055736	6.8E-10
var_112	CGGATAGCTCAGTCGGTAGAGCATCAGAC	3.64E-10	0.511958765	5.68E-09
var_110	TCCCTGTTCGGGCGCCA	8.45E-10	0.589661746	0.000000011
var_111	CCCTGTTCGGGCGCCA	2.96E-09	0.609152479	0.000000033
var_118	AAGAGGAGTTGTTTT	5.15E-09	0.574344188	5.02E-08
var_41	CCACGTTGGGCGCCA	1.33E-08	1.501943969	0.000000115
var_107	GAAGCGGGTGCTCTTATTTTT	4.32E-08	0.69382188	0.000000337
var_94	GTGTAATGGTTAGCACTCTG	9.19E-08	1.540276228	0.000000603
var_35	CATAATCTGAAGGTCCT	9.28E-08	0.669723782	0.000000603
var_65	ACCTCAGAAGGTCTCACTTT	0.000000155	0.657821804	0.000000932
var_73	ATGGTAGAGCTCTCGC	0.000000894	1.453409391	0.00000498
var_44	AGTCGGTAGAGCATC	0.00000155	0.597636377	0.00000806
var_7	TCCCCAGTACCTCCACCA	0.0000025	1.357625196	0.0000122
var_93	TAGTGGTTAGTACTCTGCGTT	0.00000465	0.683657969	0.0000213
var_39	GCCCGACTAGCTCAGTCGGTAGAGCATGGGACT	0.00000785	1.360577984	0.000034
var_3	TTAACCGAAAGGTTGGTGGTTCGAGCCCACCCAGGGACGCCA	0.0000122	0.678849094	0.0000501
var_5	AGTGGTAGAGCATTTGACT	0.0000856	0.766626221	0.000323959

var_88	CCCCGTCCGTGCCTCCA	0.0000872	0.759613212	0.000323959
var_85	TTCGTCTGTAATTTT	0.0000924	0.647075084	0.000327662
var_36	ATCTCGGTGGAACCTCCA	0.0001266	0.746116142	0.000429339
var_26	GGTTCGATTCCCGGCC	0.000155345	1.380505299	0.000504872
var_115	CTCCTGGCTGGCTCGCCA	0.000174497	0.588720748	0.000544429
var_17	GCCCGGCTAGCTCAGTCGGTAGAGCATGGGACTC	0.000233815	1.341323257	0.000701445
var_1	TCGTTTCCCGGCCAACGCACCA	0.000270644	0.763418478	0.000781861
var_9	CCCGGCACCTCCACCA	0.000338962	0.799397137	0.000944252
var_24	GGCTCCGTGGCGCAATGGA	0.000540142	1.273350881	0.001452796
var_70	GCATTGGTGGTTCAGTGGTAGAATTCTCGCCT	0.000588695	0.614061321	0.001530606
var_45	CAGTCGGTAGAGCATGAGACT	0.000632679	1.28954763	0.001591903
var_80	CCGAAGATCACGGGT	0.001402488	0.829588372	0.003418566
var_14	GCCCGGCTAGCTCAGTCGGTAGAGCATGAGACT	0.001623455	2.292244192	0.003837258
var_62	GGTAGCGTGGCCGAGCGGTCTAAGGC	0.002139559	1.220331551	0.004908399
var_27	TCCCCGTACGGGCCACCA	0.002733085	1.346561172	0.006090875
var_43	CAGTCGGTAGAGCATC	0.003289586	1.26185163	0.007127437
var_13	TCCGGGTGCCCCCTC	0.004424447	3.191802855	0.009327213
var_22	GACCGCGTGGCCTAATGGATAAGGCGTCTGACT	0.004714213	0.873846917	0.009676543
var_2	CCCACCCAGGGACGCCA	0.00591554	0.749006989	0.01183108
var_74	ATGGTAGAGAGCTCGC	0.006494284	1.189193006	0.012663853
var_121	TCCCTGGTGGTCTAGTGGTTAGGATTCGGCGCT	0.008562143	0.877138025	0.016288955
var_108	GCCCGGATAGCTCAGTCGGTAGAGCATCAGAC	0.009938899	0.829412525	0.018457956
var_20	ATCTCGCTGGGGCCTCCA	0.021628076	0.902362925	0.039232323
var_114	GGGCCAGTGGCGCAATGGATAACGCGTCTGACT	0.024422961	1.20654233	0.043054976
var_69	CCGTACGGGCCACCA	0.024839409	1.306931916	0.043054976
var_15	GCCCGGCTAGCTCAGTCGGTAGAGCATGGGACT	0.026668403	1.805607979	0.045220335
var_58	GTTTCCGTAGTGTAGTGGTCATCACGTTCGCCT	0.028792055	1.153449233	0.046912552

var_42	CCCCACGTTGGGCGCCA	0.028869263	1.201385927	0.046912552
var_113	GTCAGGATGGCCGAGT	0.03068149	1.204169763	0.048839923
var_54	GTTTCCGTAGTGTAGTGGTTATCACGTTCGCCT	0.033952529	1.142138048	0.052965945
var_99	ATCCTGCTCACAGCGCCA	0.038553972	1.169001382	0.058964899
